# Detecting Erythrocyte-Derived Extracellular Vesicles Generated from Blood Pump Flow and the Challenges Encountered

**DOI:** 10.3390/cells15070642

**Published:** 2026-04-02

**Authors:** Kylie M. Foster, Ahmed M. El Banayosy, Aly El Banayosy, Hendra Setiadi, Vivek K. Bajpai, Edgar A. O’Rear

**Affiliations:** 1 School of Sustainable Chemical, Biological and Materials Engineering, University of Oklahoma, Norman, OK 73019, USA; kylie.m.foster-1@ou.edu (K.M.F.); vkbajpai@ou.edu (V.K.B.); 2 INTEGRIS Advanced Cardiopulmonary Care, INTEGRIS Baptist Medical Center, Oklahoma City, OK 73112, USA; ahmed.elbanayosy@integrishealth.org (A.M.E.B.); hendra.setiadi@integrishealth.org (H.S.); 3 Saint Francis Heart and Vascular Institute, Tulsa, OK 74136, USA; 4 Stephenson Cancer Center, University of Oklahoma Health Campus, Oklahoma City, OK 73104, USA; 5 Department of Dermatology, University of Oklahoma Health Campus, Oklahoma City, OK 73104, USA

**Keywords:** flow cytometry, microvesicles, erythrocyte, mechanical circulatory support, LVAD, ECMO

## Abstract

**Highlights:**

**What are the main findings?**

- The availability of stains specific for porcine and bovine erythrocytes and their derived extracellular vesicles is limited. This was addressed by identifying and validating alternative labeling options.
- Perfusion of red blood cells with the CentriMag blood pump caused an increase in erythrocyte-derived extracellular vesicles with time, with bovine samples producing significantly higher levels of extracellular vesicles than porcine.

**What are the implications of the main findings?**

- Identification of alternative labels allows for the quantification of porcine and bovine erythrocyte extracellular vesicles in benchtop blood pump testing.
- Early-stage device testing could benefit from using bovine blood products, as bovine erythrocytes appear to be more sensitive to mechanical trauma in terms of extracellular vesicle production.

**Abstract:**

Utilization of a blood pump to aid in circulating a patient’s blood, otherwise known as mechanical circulatory support, is an effective and often life-saving treatment for cardiac/pulmonary failure patients, yet adverse events remain a common complication often attributed to mechanical trauma inflicted on blood components. This work specifically focuses on erythrocyte-derived extracellular vesicles (ErEVs) as a marker of this mechanical trauma as they are elevated in patients with blood pumps and have been tied to adverse events. Despite this, ErEVs are typically neglected during device development which usually includes testing with animal blood, most commonly porcine and bovine. Flow cytometry was employed to monitor ErEVs generated during a 6 h perfusion of porcine or bovine red blood cells (RBCs) in a blood circulatory loop with the CentriMag blood pump. Successful measurement meant overcoming limitations in suitable stains for the RBCs and ErEVs of the two species. Between the two species, 12 different antibodies and dyes were evaluated, including multiple glycophorin A clones, the typical human erythrocyte antigen. Only CD46 and carboxyfluorescein succinimidyl ester (CFSE) were found to successfully and reliably label porcine and bovine RBCs, respectively. With these stains, statistically significant increases for both porcine and bovine ErEVs with perfusion time were observed. Bovine erythrocytes produced significantly more ErEVs than porcine, indicating they are more sensitive to mechanical trauma and could be useful in early-stage device development. The utility of CD46 and CFSE used for porcine and bovine ErEV detection was demonstrated for in vitro pump testing with implications for physiological and pathological research with these animals.

## 1. Introduction

Therapeutic intervention for cardiac/pulmonary failure patients often involves an extracorporeal or implanted pump functioning as mechanical circulatory support. While blood pumps are an effective option, patients still experience complications such as bleeding, thrombosis, stroke, and infection [1,2,3,4]. Occurrence of adverse events is typically associated with device hemocompatibility issues often through damage to blood components as they are propelled through the pump. In early devices, the primary form of damage was hemolysis, but modern devices cause minimal levels, yet patients still experience issues [5,6,7,8]. It is understood that other forms of trauma need to be studied and measured in blood pump development to understand the full scope of remaining hemocompatibility issues in these devices.

One potential alternative damage marker for use in blood pump development is extracellular vesicles (EVs), which are membrane-enclosed nano-sized particles ranging from 50 to 1000 nm that originate from their parent cell’s membrane [9,10]. In general, EVs have garnered increasing attention for use as potential diagnostic and prognostic biomarkers in a variety of pathologies [11,12,13]. Additionally, multiple studies have shown that patients with circulatory support, such as those with compromised cardiopulmonary function, have elevated levels of circulating EVs, which are associated with adverse events [14,15,16,17] and are caused by the supraphysiologic stresses imposed with blood pumps [5,6,18,19,20,21].

As such, EVs are a logical damage marker that can be used in pump development. We are particularly interested in evaluating the release of erythrocyte-derived extracellular vesicles (ErEVs) for evaluating blood pump performance in preclinical testing and development. Of the circulating EVs in patients with devices, ErEVs tend to have higher concentrations along with platelet-derived EVs [14,17]. Additionally, our recent findings indicate that in vitro perfusion of human whole blood though a blood pump results in interactions between erythrocytes and leukocytes, likely mediated by EVs, including ErEVs [22]. These interactions have implications in the development of adverse events, specifically infection and thrombosis in patients. Interestingly, there are several studies that have evaluated platelet [5,21,23] and leukocyte EV [6,20,23,24] production from in vitro blood pump perfusion, leaving a gap in research regarding ErEV generation by pumps as reported by Pieper et al. [25]. This gap is likely a result of the challenges in detecting ErEVs in in vitro testing. Addressing this shortcoming offers potential benefits such as placing the focus more on the direct effects of shear stress rather than from platelet and leukocyte activation.

In vitro blood pump testing in blood circulatory loops (BCLs) and measurement of generated EVs typically means working with animal blood, often bovine or porcine, due to the larger volumes required [26,27,28]. Using human blood to evaluate hemocompatibility for devices designed for human patients is of course ideal, but it is often not feasible due to risk of infection, limited availability, and high financial burden [29]. As mentioned above, work has already been done evaluating both platelet [5,21,23] and leukocyte [6,20,23,24] EV production using both porcine and bovine blood, but there are no reports to our knowledge evaluating ErEV production. We sought to address this shortcoming by measuring EVs from animal red cells via flow cytometry (FCM).

Staining of porcine or bovine ErEVs proved to be a challenge to overcome. Ideally, antibodies comparable to CD235a, an erythrocyte-specific marker for glycophorin A used in several flow cytometric studies to stain human red blood cells (RBCs) and daughter EVs [14,15,19,30,31], would be used for bovine and porcine studies for ease of comparison. It appears that both porcine and bovine RBCs have analogous glycoproteins, but commercially available antibodies are limited. Of the few commercial options, we found that none reliably bound to their target [32,33,34]. To address this, alternative stains were identified for both porcine and bovine RBCs and their daughter EVs (PcEVs and BvEVs, respectively). Between the two species, 12 different antibodies, including multiple CD235a clones, were evaluated for RBC reactivity. For porcine erythrocytes, anti-CD46, which targets membrane cofactor protein (MCP), was identified. In our search, we were unable to identify any commercially available antibodies that could be used reliably to label cow erythrocytes. As such, carboxyfluorescein succinimidyl ester (CFSE), a cytosolic dye which has been used in other work to label EVs [18,31,35,36,37], was chosen to label bovine ErEVs by insertion of the dye in the cytoplasmic space of the vesicles.

In this work, we begin addressing the gap regarding FCM ErEV measurement by evaluating the utility of CD46 and CFSE for PcEV and BvEV FCM detection, respectively. Without alternatives, FCM measurement of ErEVs in device development would not be possible. Additionally, we aim to preliminarily identify which of the two species offers the greatest sensitivity to fluid stresses as assessed by ErEV production, which would be useful in identifying issues with blood pumps that other damage markers, such as hemolysis, do not reveal. The utility of both labels was examined by exposing isolated RBC suspensions to non-physiologic stresses (>10 Pa) using the CentriMag blood pump in a BCL [38]. The CentriMag is often used for extracorporeal membrane oxygenation (ECMO) treatment, a type of mechanical circulatory support [38,39], making its use in this study clinically relevant. We show that the identified labels successfully stain RBCs of each species as well as their ErEVs and are able to detect increases in ErEV concentrations with perfusion time with the CentriMag. This is a vital first step, as addressing the lack of ErEV research in bloop pump development is only possible if reliable stains are identified for use in the typical animal surrogates [26,27,28]. Additionally, these stains have potential applications in the veterinary community, such as in the study of viruses or parasites that effect the RBCs of livestock [40] and in other types of research utilizing animal blood, especially when practical/ethical concerns eliminate the use of human product.

## 2. Materials and Methods

### 2.1. Blood Collection and Preparation

As we are interested in evaluating ErEVs as a hemocompatibility marker for blood pumps, testing was performed using the common animal surrogates’ RBCs, both porcine and bovine [26,27,28]. Ovine blood is occasionally used as well, but it often produces much higher levels of hemolysis and is not considered as good a surrogate as porcine or bovine [26,27,28]. As such, ovine blood was excluded from this study. Porcine and bovine whole blood, anticoagulated with sodium citrate, were purchased from Animal Technologies (Tyler, TX, USA), a USDA-certified animal technology company which supplies biological samples for research purposes. Blood was collected and shipped overnight on ice for immediate use the day after collection. Porcine whole blood was collected from seven donor pigs (sex and age not provided) from an abattoir. Three of the donors were used to compare the use of CFSE and CD46 for labeling PcEVs (n = 3), while the remaining donors were used for assessing ErEV production between species (n = 4). Bovine whole blood was obtained from four donor cows (all female, age not provided) drawn via gravity fed venipuncture from the jugular vein using a 12-gauge needle.

For both species, blood was brought to room temperature, and then RBCs were isolated with a series of three isotonic saline washes to remove plasma proteins, lipoproteins, native EVs, platelets and leukocytes, as previously described [30]. Briefly, whole blood aliquots (40 mL) were centrifuged at 1100 *g* for 15 min in a swing bucket centrifuge, after which the buffy coat was carefully removed via Pasteur pipette and plasma removed by aspiration. Isotonic saline was then used to resuspend the RBCs prior to re-centrifugation. After each wash, any remaining buffy coat was removed. Isotonic saline for bovine was 0.9% NaCl (154 mM), while the isotonic saline used for porcine RBCs was 0.964% NaCl (165 mM), as porcine whole blood has slightly higher osmolarity [41]. After the third wash, RBCs were resuspended to 37 ± 1% packed cell volume (PCV) in a modified Ringer’s solution (147.5 mM NaCl, 4 mM KCl, 1.7 mM CaCl_2_, and 10 mM glucose, with 0.05 g/L albumin).

### 2.2. Blood Circulatory Loop Setup and Operation

**Figure 1:**
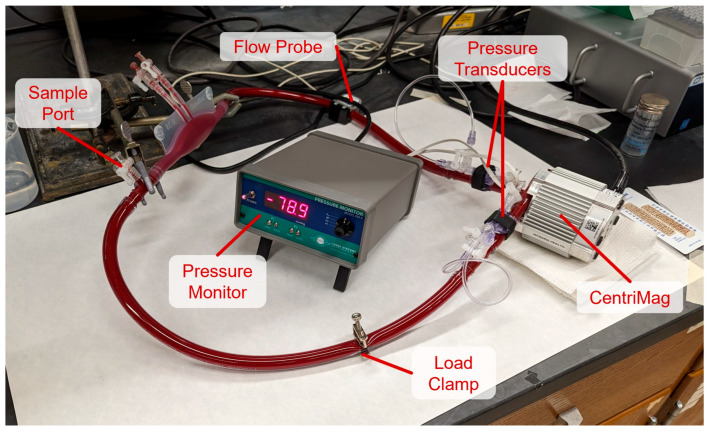
Blood circulatory loop (BCL) configuration. The CentriMag blood pump was connected with Tygon ND-100-65 hemocompatible tubing (3/8 in. IDx9/16 in. ODx43.5 in. length) to a Medtronic R-38 ECMO bladder, serving as a reservoir. Flow rate was monitored using a flow probe connected to the CentriMag console. A load of ~80 mmHg was applied to the loop using a load clamp, and inlet and outlet pressures were monitored using saline filled inline pressure transducers connected to a pressure monitor. A sample port located on the inlet side of the reservoir was used to pull samples during perfusion.

ErEVs were generated using a blood circulatory loop (BCL) connected to a CentriMag blood pump (Abbott, Abbott Park, IL, USA), due to the non-physiologic stresses imparted by the pump [38], as shown in Figure 1. The CentriMag used in testing was unused with no prior clinical exposure. The BCL design and sampling intervals were based on the American Society for Testing and Materials (ASTM) standard for in vitro hemocompatibility testing of continuous flow blood pumps (ASTM F1841-19), with minor modifications [42]. The BCL was constructed from Tygon ND-100-65 hemocompatible tubing (3/8 in. IDx9/16 in. ODx43.5 in. length, Saint-Gobain, Malver, PA, USA) with a Medtronic R-38 ECMO bladder (Medtronic, Minneapolis, MN, USA) to serve as the reservoir. Inlet and outlet pump pressure were monitored using inline pressure transducers (TranStar Disposable Transducer, Medex/Smiths Medical, Minneapolis, MN, USA) connected to a pressure monitor (Living Systems PM-4, Living Systems Instrumentation, Fairfax, VT, USA). Volumetric flow rate was monitored using the built-in flow probe on the CentriMag console. Prior to experiments, the loop was loaded and perfused with 150 mL of isotonic saline.

After the loop was rinsed and drained of isotonic saline, 150 mL of the isolated RBCs at 37% PCV was carefully loaded into the circuit. In addition to the BCL, 50 mL of the RBC suspension was set aside to serve as bulk RBCs. The loaded loop was then cleared of visible air and sealed. Washed RBCs were perfused through the loop at nominal clinical conditions of 4 L/min against an 80 mmHg afterload, applied using a Hoffman clamp, for 6 h at room temperature [22]. The loop was sampled at the start of perfusion, then hourly for the first three hours, and then again at 6 h. Prior to sampling, 1 mL of RBCs was slowly drawn and discarded to flush the sample port of stagnant RBCs, after which a 2 mL sample was collected from the loop. After sampling, 3 mL of the bulk RBCs was added back into circulation to maintain a constant volume in the BCL, and the sample port was rinsed with isotonic saline. Each sample was used to measure PCV and to conduct flow cytometry. PCV was measured using a microhematocrit centrifuge. After each experiment, the circulation loop was dismantled and all blood contacting components, including the CentriMag pump head, were soaked overnight in a Tergazyme solution (Alconox Inc., White Plains, NY, USA) and then thoroughly rinsed.

### 2.3. Characterization by Flow Cytometry

EVs are of interest in a variety of fields of study and, as such, there is an abundance of work providing guidance on EV characterization methods [43] with many reviews on the topic [37,44,45,46]. While there are several techniques available to analyze EVs such as nanoparticle tracking analysis, microscopy (fluorescent and electron), and Western blot, flow cytometry (FCM) remains one of the most common and preferred methods [47,48,49]. FCM has several advantages over these other techniques, including the ability to evaluate EVs with fast and high-throughput processing and its ability to distinguish subpopulations in heterogeneous EV samples via utilization of multiple stains targeting a variety of cell specific antigens [45,48]. These abilities are particularly advantageous for in vitro blood pump testing. The ability of FCM to distinguish subpopulations is helpful as pump evaluation is often performed with whole blood, producing a heterogeneous EV population. The speed at which samples can be analyzed is also particularly useful. Tests are lengthy, with ASTM F1841-19 recommending performing BCL tests for 6 h with samples taken hourly [42], while experiments as long as 10 h have been reported [50]. Depending on the number of pumps tested and if stagnant controls are collected concurrently, this type of testing can generate large numbers of samples for analysis. Other forms of EV analysis have time-consuming preparation steps, making it difficult/impractical to analyze samples hourly. Given the advantages of FCM, it seems to be the most suitable method of analysis for in vitro blood pump testing, although the lack of porcine and bovine RBC-specific stains complicates matters. Other forms of analysis that do not allow for subtyping or that require lengthy preparations, such as nanoparticle tracking analysis or microscopy, can provide Supplemental Data to confirm FCM results.

#### 2.3.1. Initial Alternative Stain Evaluation

To address the lack of stains to porcine and bovine RBCs, a variety of stains were first evaluated for reactivity with porcine and bovine erythrocytes. Table 1 lists the different stains tested along with the supplier, clone number, target species, amount of RBCs labeled, antibody concentrations tested, the species each stain was tested on, and the binding result. Amounts of antibodies and RBCs used were based on manufacturers’ recommendations. For each, RBCs were labeled in a total staining volume of 100 μL, supplemented with Ringer’s solution, and allowed to incubate for a minimum of 30 min. Samples were then brought to 500 μL and analyzed using flow cytometry (BD Accuri C6, Becton, Dickinson and Company, Franklin Lakes, NJ, USA). Samples were first gated on the RBC population on a forward scatter-side scatter density plot and then for positive fluorescent signal. Any antibodies that showed successful binding were further titrated using EV samples to determine optimal staining conditions for ErEV detection.

Of the antibodies in Table 1 that showed successful binding, fluorescence microscopy was used to further confirm that they reliably stain their respective species’ RBCs. A total of 1 μL of isolated RBCs at 37% PCV was stained with the appropriate label. Porcine RBCs (n = 1) were labeled with monoclonal anti-pig CD46 (0.0017 μg/μL working concentration, clone 6D8/8, BioRad, Hercules, CA, USA) conjugated to Alexa Fluor 488 (Alexa Fluor 488(Fast)-Lightning-Link, Abcam, Waltham, MA, USA). Bovine RBCs (n = 1) were labeled with CFSE (4 μM working concentration, Molecular Probes). For both, staining volumes were brought to 100 μL using Ringer’s solution, allowed to incubate at room temperature, and protected from light for 30 min before being brought to 1000 μL. Samples were washed at 1000 *g* for 5 min and brought to a final volume of 500 μL. A drop of labeled RBCs was placed on a cover slip, and cells allowed to settle and then imaged using a 60× objective (Cytation5, BioTek, Winooski, VT, USA). Each species’ RBCs were imaged on separate days and microscope settings optimized for the different fluorophores used. Background signal was subtracted using ImageJ (version v1.54p). Any antibodies that showed successful binding with FCM and fluorescence microscopy were further titrated using EV samples to determine optimal staining conditions for ErEV detection.

#### 2.3.2. Assay of Extracellular Vesicles by Flow Cytometry

Samples collected from the BCL were used to obtain EV-containing media depleted of RBCs and then used for evaluating ErEV concentrations. BCL samples were immediately processed using a two-step centrifugation procedure, established to remove RBCs in suspension without removing larger-sized ErEVs. As isolated RBCs were used for BCL perfusion, removing RBCs should leave behind only generated ErEVs in the suspending medium. Samples were first centrifuged at 500 *g* for 5 min and the supernatant collected, followed by centrifugation at 1500 *g* for 5 min to remove any remaining RBCs. The final supernatant was collected into Protein LoBind tubes (Eppendorf, Enfield, CT, USA) as the EV-containing media used for analysis. Samples were stored at 4 °C for FCM analysis the following day [51,52].

After erythrocyte removal, EV-containing samples were then used for FCM analysis with PcEVs and BvEVs identified via CD46 or CFSE, respectively. Refrigerated samples were allowed to come to room temperature for ~20 min prior to labeling. Then, 10 μL of each EV sample was labeled with the appropriate stain; CD46 (0.0033 μg/uL working concentration) for PcEVs and CFSE (4 μM working concentration) for BvEVs. One of the potential concerns with using a cytoplasmic dye as the label for BvEVs rather than a monoclonal antibody targeting a transmembrane protein (as for PcEVs) is that the difference in label type will potentially skew the EV results. To evaluate this effect, PcEV samples generated from identical BCLs (n = 3) were labeled with either CFSE or CD46 at the same concentrations as above and concentrations of detected PcEVs compared.

**Figure 2:**
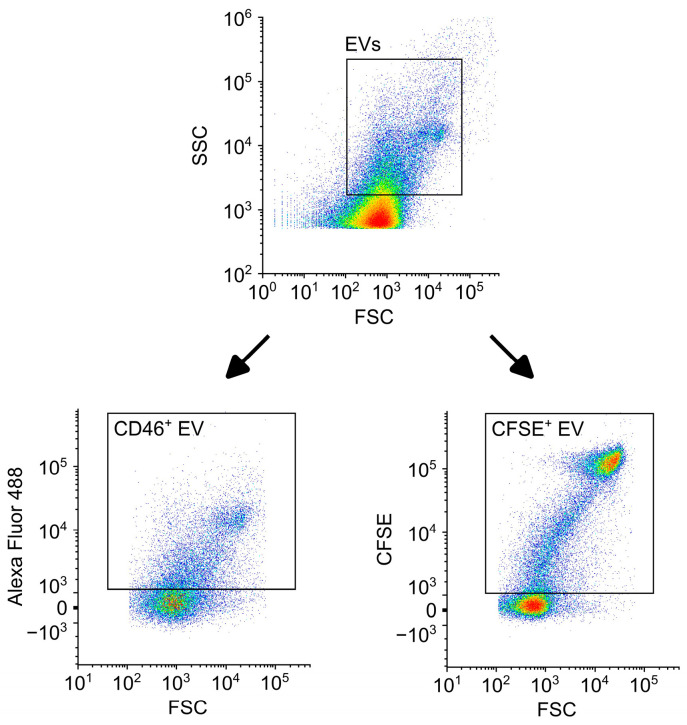
Gating schemes for flow cytometry (FCM) analysis. Events were first gated on size using a forward scatter (FSC)–side scatter (SSC) density plot. Events in the EV gate were then selected on occurrences positive for either CD46 or CFSE.

For all FCM analysis, staining volumes were brought to 100 μL using Ringer’s solution, allowed to incubate at room temperature, while protected from light for 30 min before being brought to 500 μL. For the BvEV and PcEV samples labeled with CFSE, staining was performed in Ringer’s solution without protein to limit background CFSE signal via the premature conversion of CFSE diacetate to CFSE. Spontaneous hydrolysis of CFSE diacetate into its fluorescent form, CFSE, is possible in aqueous solutions, with Ender et al. showing that the presence of protein contributes to higher levels of background signal [47,53]. After incubation, samples were then analyzed on the Cytek Northern Lightsflow cytometer (Cytek Biosciences, Fremont, CA, USA) with 2 min of sample collection at the low-collection-speed setting. The following controls were used for FCM analysis with expected results: Ringer’s solution only to evaluate background particles, Ringer’s solution with reagents (CFSE or CD46) to evaluate background fluorescence, unstained EVs to establish negative fluorescent gating and evaluate autofluorescence, and appropriate isotype controls to probe for non-specific binding. Additionally, calcium ionophore 4-bromo-A23187 (Sigma Aldrich, St. Louis, MO, USA) was added to 10 μL of bulk isolated RBCs (37% PCV) at a working concentration of 0.012 μg/μL and final incubation volume of 100 μL to induce EV formation as an EV positive control [54]. EV positive controls were incubated at 37 °C for 2 h and then processed and analyzed along with BCL samples. All FCM analysis was performed using Floreada.io. Samples were first gated by size on a forward scatter (FSC)–side scatter (SSC) density plot (Figure 2) with the EV size gate established using fluorescent polystyrene size reference beads ranging from 0.2 to 1.0 μm (Flow Cytometry Sub-Micron Particle Size Reference Kit, Thermo Fisher Scientific, Waltham, MA, USA). Polystyrene calibration beads were used to determine the lower limit of detection on the FSC-SSC density plots, below which true events are indistinguishable from background noise or debris. Due to the differences in refractive index between EVs and polystyrene beads [55], we cannot definitively say that the smallest EVs detected via FCM are 200 nm. The EV positive control was also used to help establish the EV size gate. Events in the EV gate were then gated for either CD46 or CFSE signal and ErEV concentrations calculated. Reported concentrations are ErEVs generated in the BCL, as ErEV concentrations were normalized to the start of perfusion via subtraction of t = 0 h values and thus normalized to any baseline ErEV levels. Reported changes in ErEV concentrations with perfusion time are not meant to be absolute but rather show the longitudinal trends of EV generation with exposure to mechanical trauma.

### 2.4. Nanoparticle Tracking Analysis

Nanoparticle tracking analysis (NTA) was employed to assess average particle size and particle size distribution (PSD) of ErEVs produced in the BCL. The stored EV samples used for FCM analysis were also used for NTA. The NTA unit was a ViewSizer 3000 (Horiba Scientific, Irvine, CA, USA), equipped with an AVT Manta 319C camera and blue (445 nm), green (520 nm) and red (635 nm) lasers. Samples were sequentially diluted in 0.1 μm filtered phosphate-buffered saline to yield an appropriate concentration (~125 particles/video frame) for NTA measurement. The recording settings used for the ViewSizer 3000 for EV measurements were established by Comfort et al. [56]. Briefly, they are as follows: camera frame rate 30 fps; exposure time 15 ms; 25 videos captured at 300 frames per video; blue laser power 210 mW; green laser power 12 mW; red laser power 8 mW; gain 30 dB. Software (version 2.12) analysis settings were also established by Comfort using a feature diameter of 30 pixels and the default detection threshold for a polydisperse sample.

### 2.5. Confocal Microscopy

Confocal microscopy was utilized to examine the morphology of ErEVs qualitatively. The same stored EV samples were used as for FCM and NTA. CFSE was utilized for both porcine and bovine samples. CFSE labels the entire cytoplasm, giving a more reliable indicator of size. A total of 50 μL of the ErEV media from each species was labeled, split over five aliquots. For each aliquot, 10 μL of sample was stained with 4 μM of CFSE in 100 μL staining volume supplemented using Ringer’s solution without protein. Samples were incubated for 30 min, protected from light, kept at room temperature, and brought to 1000 μL using Ringer’s solution without protein. Labeled samples were then washed twice at 200,000 *g* for 2 h at 4 °C (Ultracentrifuge CP100NX, rotor P50A3-0565, himac Eppendorf Group, Enfield, CT, USA) with the top 900 μL carefully removed [54,57]. Following the second wash, the five tubes for each species were consolidated into a single tube and brought to 1000 μL to be further concentrated via ultracentrifugation (200,000 *g*, 1 h, 4 °C) with the top 900 μL discarded. Following the method outlined in Puzar-Daminkus et al., 7 μL of EV concentrate was pipetted onto a coverslip and then sealed to a standard microscopy slide [57]. Slides were immediately imaged using a 63x/1.4 oil objective on a Leica SP8 Upright CLS/multiphoton/FLIM confocal microscope (Leica, Teaneck, NJ, USA). The lateral resolution for confocal microscopy is given by $r_{confocal}=0.4\lambda /NA$, where λ is the emission maximum for the fluorophore used, and NA is the numerical aperture of the objective [58]. This results in a resolution of ~150 nm for CFSE labeled EVs.

Images were processed using ImageJ (National Institutes of Health). Background signal was subtracted and thresholding applied to convert images to binary (black and white). Particle size analysis was performed with ImageJ, detecting particles with areas ≥ 0.03 μm^2^, equivalent to ~195 nm diameter particles, and circularity ranging from 0 to 1, similar to Puzar-Daminkus [57]. The minimum area was set to exclude detection of sub-diffraction-limited particles and ensure particles were larger than one pixel.

### 2.6. Statistical Analysis

All statistical analysis was performed in R, version 4.4.1. Normality was confirmed using both the Shapiro–Wilk test as well as examining QQ plots. Species were compared at each timepoint using Welch’s *t*-test. Additionally, significant changes with time for each BCL were evaluated with one-way repeated-measures ANOVAs with post hoc pairwise testing using the emmeans package in R with a Tukey adjustment for multiple comparisons. Comparisons between PcEVs labeled with either CD46 or CFSE were evaluated at each timepoint using Welch’s *t*-test. Significance was defined as *p* < 0.05, and values are reported as mean ± standard error of the mean (SEM) except for EV sizes from NTA which are mean ± standard deviation.

## 3. Results

### 3.1. RBC Stain Evaluation

**Figure 3:**
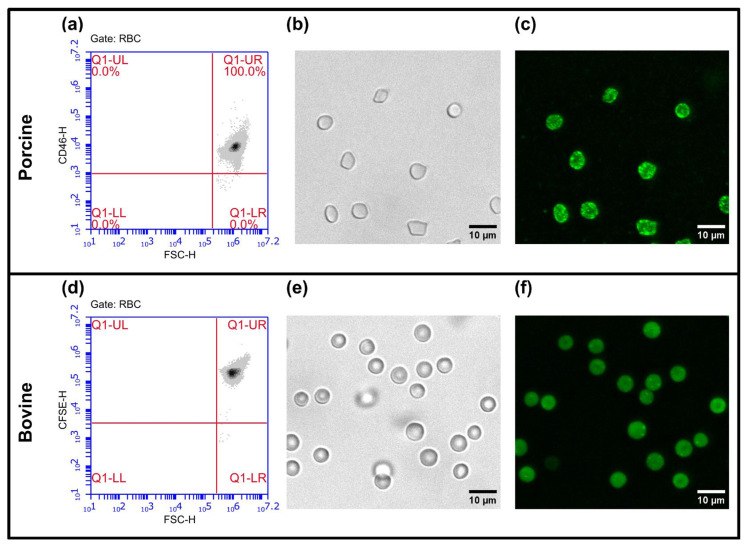
Representative flow cytometry, brightfield, and fluorescence microscopy images showing sufficient labeling of red blood cells (RBCs). All stained RBC samples (n = 1), independent of species, yield bright, uniform labeling. (**a**) A forward scatter–CD46 FCM plot gated on the RBC population shows porcine RBCs with positive CD46 signal in the Q1-UR gate. (**b**) Brightfield microscopy image of porcine erythrocytes and (**c**) corresponding fluorescence microscopy image showing bright uniform labeling of porcine RBCs with CD46–Alexa Fluor 488. (**d**) A forward scatter–CFSE FCM plot gated on the RBC population showing positive staining of bovine erythrocytes with CFSE in the Q1-UR gate. (**e**) Brightfield microscopy image of bovine RBCs and (**f**) corresponding fluorescence microscopy image showing bright uniform labeling of bovine cells with CFSE.

A variety of stains were identified and evaluated for use with porcine and bovine RBCs, outlined in Table 1. The binding of these antibodies to porcine or bovine erythrocytes was initially tested using FCM. Any stains showing positive binding via FCM were qualitatively analyzed with fluorescence microscopy. Several different monoclonal and polyclonal varieties of erythrocyte-specific CD235a were evaluated to test for possible cross-species reactivity, none of which showed successful binding. The only antibody we found that successfully bound to porcine RBCs was CD46. Successful binding of CD46 is shown via FCM results (Figure 3a) and brightfield and fluorescence microscopy (Figure 3b,c). Finding a successful option for bovine erythrocytes proved to be more difficult. None of the antibodies evaluated for bovine reactivity in Table 1 showed any positive results. As such, CFSE, a cytosolic dye which has been used in other work to label EVs, was also evaluated [18,31,35,36,37]. CFSE successfully labeled bovine RBCs, as shown via FCM (Figure 3d) and fluorescence microscopy (Figure 3e,f).

### 3.2. PcEV and BvEV Production During BCL Perfusion

**Figure 4:**
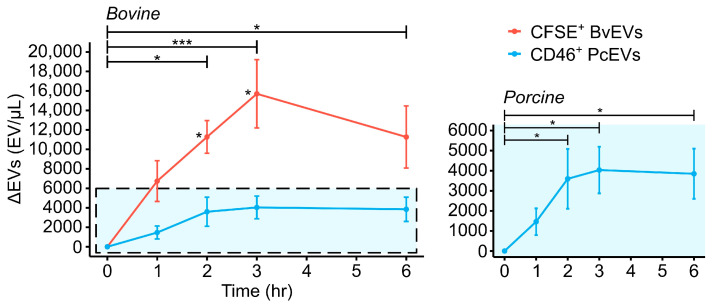
Comparisons of bovine- and porcine erythrocyte-derived extracellular vesicle (BvEV and PcEV, respectively) production, evaluated via FCM, over the course of 6 h of perfusion in the CentriMag BCL (bovine n = 4, porcine n = 4). All erythrocyte-derived extracellular vesicle (ErEV) concentrations are evaluated as changes from the start of perfusion. ErEVs positive for CFSE or CD46 for BvEVs and PcEVs, respectively, increased with perfusion time using the CentriMag for both species. The inset shows PcEV concentrations separately from BvEV data so that longitudinal trends can be more easily observed. BvEV production was significantly higher than PcEV concentrations at 2 h and 3 h only (*p* < 0.05). ErEV concentrations increased significantly over the start of perfusion beginning at 2 h for both bovine and porcine samples (as shown in the inset) (*p* < 0.05). Data is presented as mean ± SEM. Statistical differences between species were assessed using Welch’s *t*-test and differences in perfusion time using a one-way repeated-measures ANOVA with emmenas post hoc testing with a Tukey adjustment, * *p* < 0.05, *** *p* < 0.001.

The ability of CD46 and CFSE to detect increases in ErEV concentration with perfusion time was evaluated for their respective species, and preliminary comparisons were made. ErEVs were generated by perfusion of isolated RBCs in a blood circulatory loop using the CentriMag blood pump (porcine n = 4, bovine n = 4) and analyzed via FCM analysis. Raw FCM data can be found in Supplemental Table S1. Baseline ErEV concentrations, generated during the RBC isolation process, were 5793 ± 526 PcEV/μL and 9986 ± 1177 BvEV/μL. Increases in ErEV concentrations over the start of perfusion at 6 h were 3848 ± 1247 EV/μL and 11,270 ± 3190 EV/μL for CD46^+^ PcEVs and CFSE^+^ BvEVs, respectively (Figure 4). CFSE^+^ BvEVs and CD46^+^ PcEVs increased significantly from the start of perfusion beginning at 2 h (*p* < 0.05 by one-way repeated-measures ANOVA with emmeans post hoc testing with Tukey adjustment). The level of BvEVs produced was an order of magnitude higher than that measured for PcEVs, as shown in Figure 4. Perfusion of bovine RBCs generated significantly higher levels of ErEVs compared to perfusion of porcine RBCs at 2 and 3 h only (*p* < 0.05 by Welch’s *t*-test).

**Figure 5:**
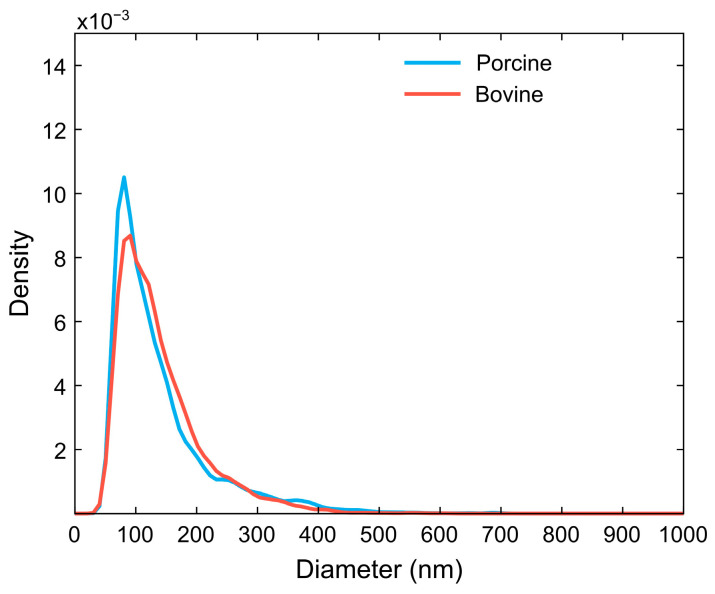
Representative particle size distributions taken from 3 h BCL samples for generated BvEVs and PcEVs.

NTA measurements were also performed to evaluate mean EV diameters and size distribution (Figure 5). EV sizes and size distributions were found to be similar to that reported in the literature (~150–200 nm) for EVs in general and for ErEVs generated due to shear exposure [59,60,61] with mean diameters of 133 ± 11 nm and 144 ± 13 nm for porcine- and bovine-derived ErEVs, respectively. Mean diameters are reported as mean ± SD.

**Figure 6:**
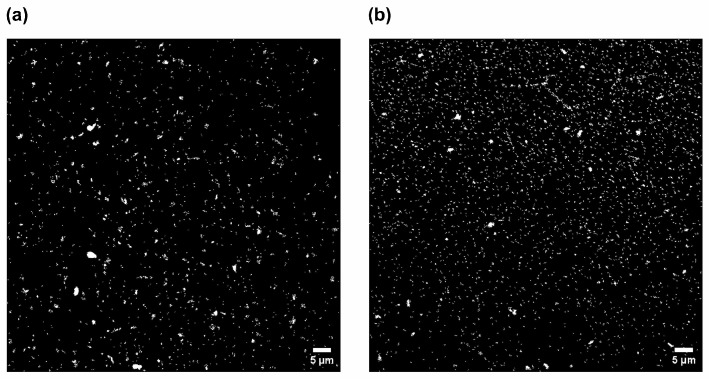
Binary masks from fluorescent confocal microscopy images of CFSE-labeled (**a**) PcEVs and (**b**) BvEVs generated from 6 h of BCL perfusion.

Particle size analysis was also performed using fluorescent confocal images and ImageJ. Binary masks showing the detected particles are shown in Figure 6. Median particle areas for porcine and bovine ErEVs were 0.057 μm^2^ and 0.058 μm^2^, corresponding to an equivalent diameter of 269 nm and 272 nm, respectively. These diameters are in alignment with PSDs in Figure 5 for both bovine and porcine BCLs.

### 3.3. Monoclonal Antibody Versus Cytosolic Dye for PcEV Detection

As CFSE is a generic cytosolic dye rather than a monoclonal antibody like CD46 or CD235a which is commonly used for staining human RBCs and ErEVs, we sought to evaluate the utility of CFSE for PcEV detection and compare it to using CD46. These results aid in interpreting the results in Figure 4, as the difference in stain type could impact the overall concentrations of ErEVs detected. A secondary set of porcine BCLs (n = 3) were run for this purpose, among which PcEVs were labeled with both CFSE and CD46. Increases in PcEVs normalized to the start of perfusion after 6 h were 692 ± 2835 CFSE^+^ PcEV/μL and 2119 ± 861 CD46^+^ PcEV/μL. There were no significant differences between CFSE- or CD46-labeled PcEVs at any timepoint (n = 3, *p* ≥ 0.516 at every timepoint by Welch’s *t*-test), indicating that either label provides similar PcEV concentrations.

**Figure 7:**
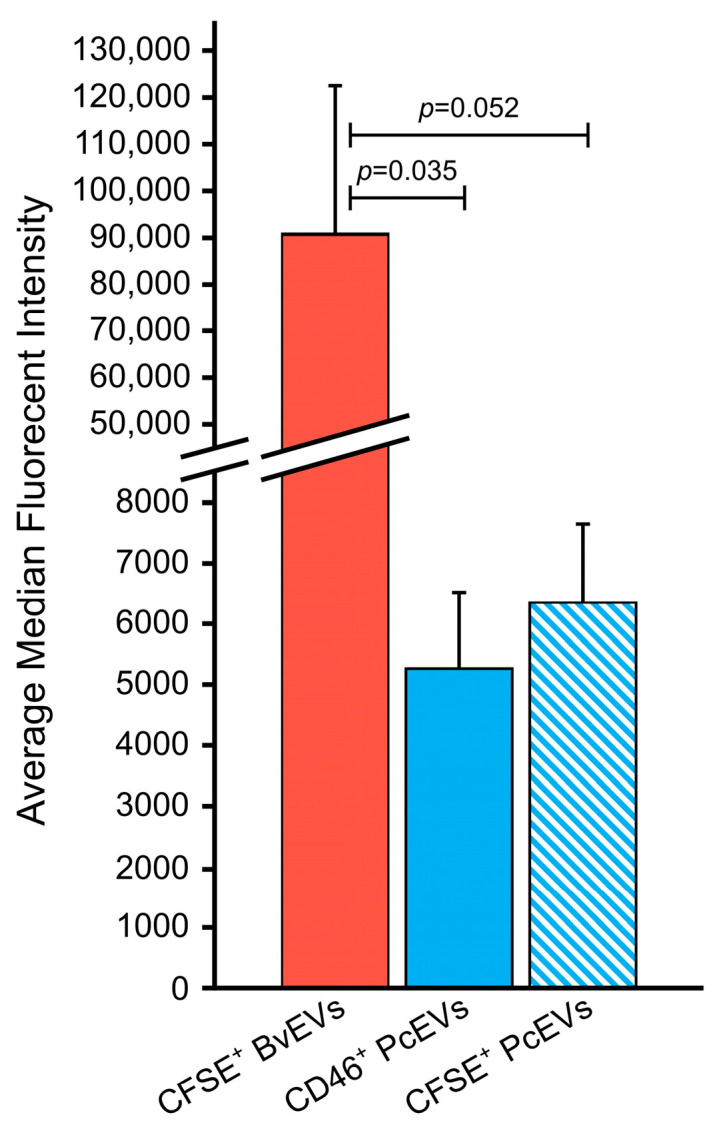
Average median fluorescent intensity at 3 h for CFSE^+^ BvEVs (n = 4), CD46^+^ PcEVs (n = 4), and CFSE^+^ PcEVs (n = 3). Median CFSE BvEV intensity was significantly larger than CD46 PcEV median intensity (*p* = 0.035) and appears to be larger than CFSE PcEV median intensity although not statistically significant (*p* = 0.052). Median fluorescent intensity for PcEVs for both CFSE and CD46 was not significantly different. Statistical differences were probed using a one-way ANOVA with emmeans post hoc testing with a Tukey adjustment.

Average median fluorescent intensities for CFSE^+^ BvEVs, CFSE^+^ PcEVs, and CD46^+^ PcEVs were compared at 3 h, as a representative timepoint (Figure 7). Median CFSE fluorescent intensity was far greater for BvEVs than for PcEVs, approaching significance (*p* = 0.052 by one-way ANOVA with emmeans post hoc testing with Tukey adjustment). CFSE BvEV median intensity was significantly larger than CD46 PcEV median intensity (n = 4) (*p* = 0.035 by one-way ANOVA with emmeans post hoc testing with Tukey adjustment).

## 4. Discussion

The use of flow cytometry for experiments with animal blood can mean facing and overcoming technical constraints. For example, Ringl et al. found certain combinations of antibodies could inhibit CD8β detection on porcine lymphocytes expressing CD8α/β [62]. The issue exists in part because the extent of the literature on animal blood is far less than that for work with human blood. Given the challenges in finding available RBC-specific stains for porcine and bovine erythrocytes and consequently their ErEVs, we sought to evaluate alternative stains for their detection. Identifying alternative stains is specifically beneficial for use in blood pump testing as these two species are often used in place of human blood. Additionally, a preliminary assessment comparing ErEV production between porcine and bovine RBCs was performed. Previous species comparison work for device testing has been performed but has primarily focused on hemolysis, with porcine or bovine typically recommended as adequate surrogates to human [26,27,28]. It is not clear if this recommendation on surrogate species for hemolysis will hold for other measures of trauma such as ErEV release. Thus, addressing the challenges surrounding ErEV detection in these species and performing preliminary comparisons is paramount for furthering the development of blood pump in vitro evaluation.

### 4.1. Alternative Label Identification and Evaluation

The typical choice for an RBC marker is the erythrocyte-specific transmembrane protein, glycophorin A (GPA). GPA is highly abundant (~10^6^ copies per human RBC) [63] and frequently used for detection of human RBCs and their daughter EVs using FCM via the antibody CD235a [19,30,31]. As it appears that porcine and bovine erythrocytes have analogous glycoproteins to human GPA [32,33,34], a variety of anti-CD235a antibodies, both monoclonal and polyclonal, were evaluated for reactivity with both porcine and bovine RBCs (Table 1). This included multiple different clones from a variety of suppliers, two of which were identified as having reactivity with bovine GPA. Unfortunately, none of these antibodies successfully stained porcine or bovine RBCs, including those supposedly reactive to bovine GPA. The lack of cross-reactivity is attributed to porcine GPA having poor homology with human GPA in the extracellular domain of the glycoprotein, and bovine GPA being heavily glycosylated (~80% carbohydrates) with speculated distinct structural differences to human GPA [33,64]. Given the lack of species-specific or even cross-reacting antibodies for porcine or bovine GPA, alternative labels were chosen and evaluated.

For identifying possible markers for use in porcine RBC detection, the work of Dawson and Lunney proved useful [32]. They reported a comprehensive list of cluster of differentiation (CD) markers for porcine, human, and murine models. Their work showcases the lack of available options, as there are only 4 CD markers identified for porcine erythrocytes with upwards of 20 CD markers identified for human and murine RBCs. Anti-CD46, targeting membrane cofactor protein (MCP), was reported to label porcine erythrocytes. MCP is a transmembrane protein which inhibits complement activation on host cells [65] and is present on all porcine circulating cells. While the number of copies of MCP per porcine RBC is unknown, it has been reported that MCP is highly and uniformly expressed on porcine RBCs, with Pérez De La Lastra et al. showing that porcine erythrocytes stained for MCP exhibited intense and uniform staining [32,66]. Similarly, we show that porcine RBCs labeled with CD46–Alexa Fluor 488 are uniformly labeled (Figure 3a–c).

Finding a suitable stain for bovine RBCs proved to be more difficult. Aside from the anti-CD235a antibodies (specific to both human and bovine GPA), anti-CD233 targeting band 3, another common RBC transmembrane protein [63], was also tried. Other less obvious choices were also evaluated, including anti-CD44, which was suggested to be present on bovine RBCs by a commercial supplier; however, a further search of the literature found that it is only expressed on early bovine erythroid cells [67]. As CD46 was successful for porcine RBCs, it was also evaluated for bovine with no success. In the absence of identifying suitable antibodies for bovine erythrocytes, CFSE, a cytosolic dye which has been used in other work to label EVs, was chosen to label bovine RBCs and BvEVs [18,31,35,36,37]. CFSE was chosen rather than a lipophilic membrane dye, such as one from the PKH family, as these lipophilic dyes produce artifacts of similar size and fluorescence to labeled EVs [35]. As shown in Figure 3d–f, CFSE readily labels the cytoplasm of bovine erythrocytes.

While we showed that both CD46 and CFSE uniformly stain their respective species’ RBCs, we assume that they will label ErEVs at comparable device sensitivity. It is known that both cytosolic and plasma membrane proteins of the parent cell tend to be abundant in EVs [10], suggesting that this assumption and the alternative stain selection are appropriate.

### 4.2. Interspecies ErEV Comparisons and Relevant Complicating Factors

Interspecies comparisons are necessary to identify if there is a better choice between porcine or bovine when evaluating ErEV production and cell injury in device testing. It should be noted that future work might compare ErEV production to that observed for humans, so that it is known which species best predicts human ErEV production. Such interspecies comparisons are complicated by the lack of commercially available erythrocyte-specific antibodies for both porcine and bovine RBCs. Nonetheless, this work provides an important first examination using alternative stains to compare performance with the typical surrogates used for blood pump testing during early-stage device development.

A preliminary analysis of ErEV production between species revealed some potential differences. The FCM results in Figure 4 show that BvEV generation was significantly higher than PcEVs at 2 and 3 h, by approximately one order of magnitude. The lack of significance at 6 h is likely due to an anomalous data point lowering the mean and increasing the standard error in the bovine population. It should be noted that the apparent difference in PcEV and BvEV production could be an effect of the differences in stains used (antibody versus cytosolic dye) and associated binding constants and, as such, comparisons between the two species in terms of susceptibility to EV formation should be considered carefully.

An additional set of porcine BCLs (n = 3) in which generated PcEVs were labeled with CFSE or with CD46 were performed to help determine if the species differences in Figure 4 are valid. When comparing CFSE^+^ PcEVs and CD46^+^ PcEVs from this additional set of BCLs, one finds no significant differences at any timepoint in contrast to the results in Figure 4. Additionally, comparison of CFSE^+^ BvEVs to CFSE^+^ PcEVs produces similar results, with significant differences at 2 and 3 h. The confocal images of CFSE-labeled PcEVs and BvEVs in Figure 6 also support the FCM results in Figure 4, with a higher density of detected BvEVs (3226 particles) compared to PcEVs (1571 particles). This provides confidence that the use of CFSE is not inflating BvEV concentrations to such an extent that they are significantly higher than CD46^+^ PcEVs and that the species differences in Figure 4 are legitimate. Interestingly, this is distinct from the trends observed in past species comparison experiments measuring hemolysis. Hemolysis comparisons often show that bovine and porcine produce similar levels, on par or slightly less than that seen in human, while our data suggests BvEV production is far greater than that of PcEVs [26,27,68]. Chan et al. also report differing trends between species, dependent on which damage marker is being evaluated [26]. The difference in trends makes it difficult to predict what might be expected for human ErEV production in similar setups, emphasizing the importance of evaluating species-dependent differences for all damage markers of interest in blood pump development. We plan to perform further BCL tests using human RBCs to see where they fall relative to ErEV production observed for bovine and porcine RBCs.

Interestingly, ErEV production in both species appears to plateau after 3 h (Figure 4), despite the overall differences in concentrations. One possible explanation would be the presence of an RBC subpopulation that is more susceptible to ErEV release under the relatively low magnitudes of stress imparted by the CentriMag [20,69]. The susceptible population would initially release EVs, but with continued perfusion this population and/or their ability to produce EVs is depleted. This theory seems unlikely as the susceptible population would be less than 0.5% of all RBCs, using a conservative assumption that each ErEV comes from a single cell. A more likely explanation would be that the plateauing effect is related to the resolution limit (~200 nm) of the flow cytometer, as established by polystyrene calibration beads. Particle size distribution analysis of EVs from the literature shows that average EV diameters are ~150–200 nm [59,60] with a large portion of the population smaller than 200 nm, of which our NTA data is in agreement [35,59,70,71]. Median diameters found from confocal microscopy are similar but subject to the limitations of optical microscopy. Considering that the polystyrene beads scatter more light than EVs of similar size, due to a higher refractive index [55], it is unlikely that FCM detects this large portion of EVs. We hypothesize that with continued perfusion, ErEV production skews towards sub 200 nm particles which are not being detected via FCM, creating this plateauing effect. Further evaluation of this phenomenon is outside the scope of this work, as the primary focus is on evaluating the utility of stains for use in FCM detection of ErEVs. We plan to explore the plateauing phenomenon further through additional analysis of future work.

An alternative solution to the lack of analogous antibodies would be to use CFSE to label all ErEVs, regardless of the species of interest, with the added downside of forgoing any cell specificity that antibodies can provide. Comparisons of CFSE^+^ PcEVs to CFSE^+^ BvEVs were also evaluated to examine the utility of using CFSE for both BvEV and PcEV detection. Comparing ErEV concentrations at each timepoint yields similar results to Figure 4, with significant differences at 2 and 3 h only between CFSE^+^ BvEVs (n = 4) and CFSE^+^ PcEVs (n = 3) (*p* < 0.05 by Welch’s *t*-test). Despite this, we believe it is more beneficial to use antibodies when available, especially as it appears that the use of CFSE does not significantly skew the measured concentrations. One reason for this is that antibodies can potentially provide some level of parent cell specificity (antibody-dependent), whereas CFSE cannot. Additionally, our data shows that CFSE appears to elevate the variability in concentration measurements via FCM. Our work indicates that perfusion of porcine RBCs does in fact produce smaller levels of ErEVs than perfusion of bovine erythrocytes, so the added level of variability might make it difficult to detect significant results.

### 4.3. Limitations

The primary limitation/challenge of this work was the inability to procure analogous stains for detecting PcEVs and BvEVs compared to the typical erythrocyte marker used for human ErEVs. This resulted in identifying alternative stains and examining their utility in detecting ErEV changes via FCM with BCL perfusion using the CentriMag. The need for alternative stains introduces some additional challenges.

First, both CFSE and CD46 pose challenges for parent cell specificity in FCM measurements using whole blood. Antibodies to CD46 will label all types of porcine circulating cells [32], including endothelial, and their EVs, and CFSE will label any membrane enclosed cell/vesicle. The use of these stains leaves some ambiguity in the exact origin of the detected EVs and whether the outlined protocol produces and detects ErEVs. As a check, a single run utilizing human RBCs and an antibody to the erythrocyte-specific marker CD235a were shown to yield similar trends with increasing human ErEVs with perfusion time. This indicates that our FCM protocol is sufficient to produce and detect increases in ErEVs.

For some experiments with CFSE, it is conceivable that the specificity issue could be bypassed by isolating and labeling erythrocytes with subsequent whole-blood reconstitution, although this would only be realistic for smaller volume studies. A cursory search of the literature and major suppliers shows that there are commercially available antibodies specific for bovine and porcine leukocytes and platelets that can be used in combination with CFSE or CD46 to identify EV subpopulations in whole blood [21,24,26,32]. Leukocyte and platelet EVs can be directly identified via double staining with CFSE/CD46 and a parent cell-specific label. Any remaining EVs, only labeled with CFSE/CD46, are likely erythrocyte EVs but could also be endothelial-derived [32]. For BCL testing, any increases in this CFSE/CD46-only population would be attributed to RBCs as (1) they are the most abundant cell and (2) endothelial EV production would not be expected in in vitro testing. While this approach is not ideal, the lack of erythrocyte-specific antibodies for porcine or bovine RBCs limits the options available for FCM analysis. The identified alternatives we propose provide a way to begin examining all EV types through this negative staining approach until better-suited labels are widely available.

A secondary concern in using these alternative stains is that they label EVs in different ways and likely have differing affinities for their targets. CD46 binds to the surface antigen MCP, and CFSE diffuses through the membrane and labels the cytosolic space. It is conceivable that similar-sized ErEVs could have a large enough volume to be detected using CFSE, while not having enough surface antigen expression to be detected with CD46, thereby potentially confounding the interpretation of our results. This fact alone makes it difficult to compare PcEV and BvEV concentrations directly. We attempted to address this concern by comparing PcEVs labeled with either CD46 or CFSE and found that both dyes provide similar results. Additionally, we compared CFSE^+^ BvEV to CFSE^+^ PcEVs and found similar results to those reported in Figure 4. While this does provide confidence in our results and indicates that the observed species differences in Figure 4 are genuine, the differing label types are still a factor that must be kept in mind during interpretation.

## 5. Conclusions

Alternative labels for use in detecting BvEVs and PcEVs via FCM were identified and were shown to reliably stain their respective species, RBCs and ErEVs. More importantly, these stains were found to be effective in assessing mechanical trauma in a blood pump by ErEV measurement. The lack of commercially available antibodies targeting bovine or porcine GPA, the typical erythrocyte antigen, requires the use of alternative labels in order to evaluate ErEV production in mechanical circulatory support devices like blood pumps. This is an important first step in addressing the lack of ErEV research for device development, as these types of studies are only possible if adequate stains are identified. Additionally, there is potential for wider use of these results across the veterinary community as well as in other types of research utilizing animal models/products.

While it is difficult to say conclusively that bovine RBCs produce more ErEVs than porcine RBCs due to the study limitations, the data reported here indicates that this is likely true considering the large difference between BvEV and PcEV concentrations (and because PcEVs labeled with both dyes result in comparable numbers as well as similar results when CFSE^+^ PcEVs are compared to CFSE^+^ BvEVs). This indicates that bovine RBCs are more sensitive to trauma in regard to ErEV production and could be useful in early-stage device development. Future work should include comparing BvEV and PcEV production to that observed by human RBCs in BCL setups as well as supplemental ErEV analysis to corroborate results and trends.

## Figures and Tables

**Table 1:** Antibodies and dyes evaluated for bovine and porcine reactivity.

Antibody	Clonality	Target Species	Supplier	RBC Amounts	Antibody Amounts	Species Tested	Result ^†^
CD44- FITC	Monoclonal IL-A118	Bovine	BioRad	10^6^ Cells	1–4 μg	Bovine	✗
CD46- Alexa Fluor 488	Monoclonal 6D8/8	Porcine	BioRad	10^6^ Cells	0.17–0.83 μg	Bovine Porcine	✗ ✓
CD46- FITC	Monoclonal MEM-258	Human, Bovine	Invitrogen ^1^	10^6^ Cells	0.15–0.6 μg	Bovine	✗
CD233- FITC	Monoclonal BRIC 6	Human	American Research Products ^2^	10^6^ Cells	2–4 μL *	Bovine	✗
CD235a- FITC	Monoclonal CLB-ery-1 (AME-1)	Human	Invitrogen	10^6^ Cells	2 μg	Bovine	✗
						Porcine	✗
CD235a- PE	Monoclonal HIR2	Human	Invitrogen	10^6^ Cells	10–20 μL *	Porcine	✗
CD235a- PE	Monoclonal HIR2	Human	BioLegend ^3^	10 μL	1–2 μg	Porcine	✗
CD235a- PE	Monoclonal GPHN02	Human, Bovine	Biotium ^4^	10^6^ Cells	1–5 μg	Bovine	✗
CD235a- APC	Monoclonal HIR2(GA-R2)	Human	Invitrogen	10^6^ Cells	0.015 μg	Porcine	✗
CD235a- Alexa Fluor 488	Monoclonal SPM183	Human, Bovine	MyBioSource ^5^	10^6^ Cells	1–10 μg	Bovine	✗
CD235a- FITC	Polyclonal	Human	Invitrogen	10 μL	0.25–2 μg	Bovine	✗
						Porcine	✗
CFSE	NA	NA	Molecular Probes ^6^	10^6^ Cells	5 μM	Bovine	✓
Ter-119- FITC	Monoclonal Ter-119	Mouse	Invitrogen	10^6^ Cells	0.5–1.0 μg	Bovine	✗
						Porcine	✗

* Antibody concentration not disclosed. **^†^** ✗ indicates unsuccessful RBC binding; ✓ indicates successful RBC binding. ^1^ Carlsbad, CA, USA; ^2^ Waltham, MA, USA; ^3^ San Diego, CA, USA; ^4^ Fremont, CA, USA; ^5^ San Diego, CA, USA; ^6^ Eugene, OR, USA.

## Data Availability

The original contributions presented in this study are included in the article/Supplemental Table S1. Further inquiries can be directed to the corresponding author.

## Supplementary Materials

The following supporting information can be downloaded at: https://www.mdpi.com/article/10.3390/cells15070642/s1, Table S1: Raw Flow Cytometry Data.

## Abbreviations

The following abbreviations are used in this manuscript:

ASTM	American Society for Testing and Materials
BCL	Blood Circulatory Loop
BvEV	Bovine Erythrocyte-Derived Extracellular Vesicle
CD	Cluster of Differentiation
CFSE	Carboxyfluorescein Succinimidyl Ester
ECMO	Extracorporeal Membrane Oxygenation
ErEV	Erythrocyte-Derived Extracellular Vesicle
EV	Extracellular Vesicle
FCM	Flow Cytometry
FSC	Forward Scatter
GPA	Glycophorin A
MCP	Membrane Cofactor Protein
NTA	Nanoparticle Tracking Analysis
PcEV	Porcine Erythrocyte-Derived Extracellular Vesicle
PCV	Packed Cell Volume
PSD	Particle Size Distribution
RBC	Red Blood Cell
SEM	Standard Error of the Mean
SSC	Side Scatter
